# Supplementary material for: The effect of intrapartum prolonged oxygen exposure on fetal metabolic status: secondary analysis from a randomized controlled trial
Source: Front Endocrinol (Lausanne). 2023 Jun 27;14:1204956. doi: 10.3389/fendo.2023.1204956 (PMC10335765; doi:10.3389/fendo.2023.1204956)
Supplement: Supplementary file 3 [file DataSheet_3.zip › Result-X101SC21092977-Z01-J001-B1-42/4.MetDiffAnalysis/O.vs.A/O.vs.A_pos_heatmap_detail.pdf]

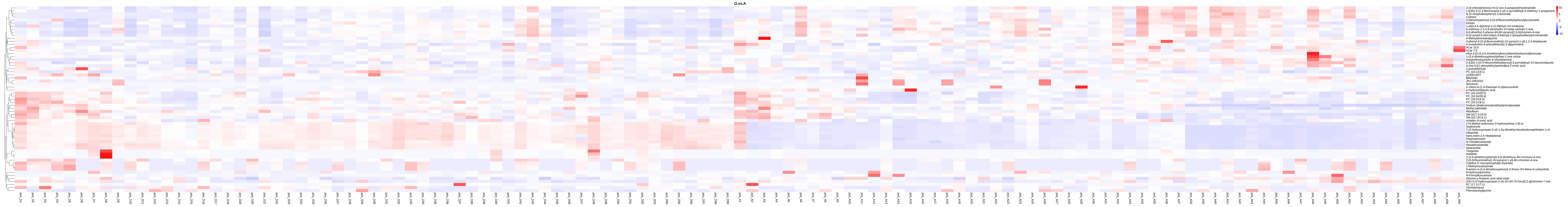
O.vs.A
10
5
0
-5
-10
2-(4-chlorophenoxy)-N-(2-oxo-3-azepanyl)nicotinamide
1-[(3S)-3-(1,3-Benzoxazol-2-yl)-1-pyrrolidinyl]-3-methoxy-1-propanone
N-(2-morpholinophenyl)-2-furamide
Cytisine
3-(dimethylamino)-2-[3-(trifluoromethyl)phenyl]acrylonitrile
Kinetin
1-allyl-4,5-diphenyl-2-(2-thienyl)-1H-imidazole
6-methoxy-2,3,4,9-tetrahydro-1H-beta-carbolin-1-one
8,8-dimethyl-2-phenyl-4H,8H-pyrano[2,3-h]chromen-4-one
N-[2-acetyl-5-(tert-butyl)-3-thienyl]-2-(propylsulfanyl)nicotinamide
4-Methylaminoantipyrine
3-phenyl-5-[3-(trifluoromethyl)-1H-pyrazol-1-yl]-1,2,4-thiadiazole
4-morpholino-6-phenylthieno[2,3-d]pyrimidine
ACar 15:0
ACar 7:0
ethyl 4-[2-(3,4,5-trimethoxybenzylidene)hydrazino]benzoate
1-(3,4-dimethoxyphenyl)ethan-1-one oxime
morpholino(quinolin-6-yl)methanone
2-{(3S)-1-[4-(Trifluoromethyl)benzyl]-3-pyrrolidinyl}-1H-benzimidazole
4-oxo-4-[(1-phenylethyl)amino]but-2-enoic acid
Cuminaldehyde
PC (18:1/18:1)
(±)5(6)-EET
Biliverdin
JNJ-1661010
Serotonin
2-chloro-6-(1,4-thiazinan-4-yl)benzonitrile
α-Hydroxyhippuric acid
PC (18:1e/20:2)
PC (16:2e/26:4)
PC (19:2/18:3)
PC (19:1/19:1)
Sodium [dodecanoyl(methyl)amino]acetate
Methyl palmitate
Riboflavin
SM (d17:1/15:0)
SM (d17:0/14:1)
octadec-9-ynoic acid
17α-Methyl-androstan-3-hydroxyimine-17β-ol
Isophorone
7-(2-hydroxypropan-2-yl)-1,4a-dimethyl-decahydronaphthalen-1-ol
Oleamide
trans,trans-2,4-Heptadienal
Fenpropimorph
N-Tetradecanamide
Hexadecanamide
Stearamide
Tangeritin
Nobiletin
2-(2,6-dimethoxyphenyl)-5,6-dimethoxy-4H-chromen-4-one
2-[3-(trifluoromethyl)-1H-pyrazol-1-yl]-4H-chromen-4-one
Cytidine 5'-monophosphate (hydrate)
1-Methylnicotinamide
6-amino-4-(3,4-dimethoxyphenyl)-2-thioxo-2H-thiine-5-carbonitrile
8-Hydroxyquinoline
N-Formylkynurenine
Dihomo-γ-linolenic acid ethyl ester
(2S)-2-(2-hydroxypropan-2-yl)-2H,3H,7H-furo[3,2-g]chromen-7-one
PC (17:1/17:1)
Ohmefentanyl
Phenylacetylglycine
pos_A58
pos_A59
pos_A60
